# Conscious sedation with dexmedetomidine for implantation of a phrenic nerve stimulator in a pediatric case of late-onset congenital central hypoventilation syndrome

**DOI:** 10.1186/s40981-017-0117-2

**Published:** 2017-08-31

**Authors:** Keiko Hirooka, Kotoe Kamata, Shiro Horisawa, Minoru Nomura, Takaomi Taira, Makoto Ozaki

**Affiliations:** 1 Department of Anesthesiology, Tokyo Women’s Medical University, 8-1 Kawada-cho, Shinjuku-ku, Tokyo 162-8666 Japan; 2 Department of Neurosurgery, Tokyo Women’s Medical University, 8-1 Kawada-cho, Shinjuku-ku, Tokyo 162-8666 Japan

**Keywords:** Congenital central hypoventilation syndrome, Diaphragm pacing, Postoperative sedation, Dexmedetomidine

## Abstract

Patients with congenital central hypoventilation syndrome (CCHS) develop alveolar hypoventilation resulting from a failure of central ventilatory control. Late-onset CCHS (LO-CCHS), which may be precipitated by severe respiratory infection or exposure to sedatives or general anesthesia, presents after the neonatal period. Since CCHS patients require lifelong mechanical-assisted ventilation, in western countries, diaphragm pacing is used to provide adequate alveolar ventilation and oxygenation during rest and daily activities. The main anesthesia-related concern regarding CCHS is postoperative respiratory failure or apnea, and anesthetic agents should be minimized to avoid further respiratory depression after surgery. A 5-year-old girl with LO-CCHS was referred to our hospital for implantation of a phrenic nerve stimulator for diaphragm pacing. Respiratory infection triggered the need for permanent nocturnal ventilator support at age 3 years and tracheotomy was performed at age 4 years. Repeated self-dislodgement of the ventilator tube led to hypoxic ischemic encephalopathy. The patient was thought to require mechanical ventilation under minimum sedation and pain management during the early postoperative period. The co-administration of dexmedetomidine and morphine provided effective conscious sedation with protection of the surgical site and without adverse events. She was discharged from the intensive care unit with a home ventilator at 3 days post-operation.

## Background

Congenital central hypoventilation syndrome (CCHS) is characterized by the lack of adequate autonomic control of respiration with decreased sensitivity to hypercapnia and hypoxia in the absence of neuromuscular or lung disease or an identifiable brainstem lesion [1]. In the latest Japanese survey, 22 of 37 CCHS patients survived without further disability, 8 survived with disability, and 7 died [2]. However, there are no data on the prevalence of late-onset CCHS (LO-CCHS), which refers to patients who are diagnosed with CCHS after 28 days of age [3]. Since respiratory dysfunction is pathognomonic of CCHS, when such patients must undergo surgery, relatively short-acting sedatives are preferred to reduce the incidence of postoperative apnea. Recently, CCHS has been recognized as a variant of generalized autonomic nervous system dysregulation because complications extend to various organ systems [4]. These presentations involve cardiac rhythm disturbance, transient asystole, decreased heart rate variability, diminished cardiovascular compensatory mechanisms, altered pain perception, and abnormal thermoregulation [5]. In our case, the presence of sympathetic nervous system tumor represented further clinical evidence of generalized autonomic nervous system dysregulation; 5–10% of CCHS cases have a neurocristopathy [6]. Central nervous system disorders in this population, including neurodevelopmental defects, may affect the decision-making process regarding anesthesia.

Patients with CCHS require lifetime ventilation support to avoid pulmonary hypertension and cor pulmonale, which results from a prolonged period of hypoxia. Diaphragm pacing, as an alternative to a mechanical ventilator that can eliminate the need for positive pressure ventilation (PPV), has been shown to improve quality of life and extend survival in patients with advanced respiratory muscle weakness [7]. Nevertheless, this surgical treatment has seen only limited use in eastern Asian countries, including Japan; in fact, a device that is specially made for phrenic nerve stimulation can only be obtained by private import. There has been only one report of a CCHS patient who underwent diaphragmatic pacing in Japan, but the patient details were not provided [2]. As Taira et al. proposed, diaphragm pacing by stimulation of the phrenic nerve with a spinal cord stimulator could be a substitute for such a device [8].

In this report, we present a pediatric case of LO-CCHS who underwent implantation of a phrenic nerve stimulator for diaphragm pacing. Due to her history of repeated self-dislodgement of a ventilator tube, which was associated with her mental retardation, postoperative sedation that protected the surgical site without severe respiratory depression was considered to be required during the early postoperative period.

## Case presentation

### Pre-operative course

A 5-year-old, 17-kg girl, who had been diagnosed elsewhere as LO-CCHS and who had required nocturnal PPV through a tracheostomy since the age of 4 years, was referred to our hospital for implantation of a phrenic nerve stimulator for diaphragm pacing. She was born at 40-week gestation and weighed 3170 g without fetal distress. She did not show any developmental disability until the age of 2 years, when irregular breathing during sleep was recognized. Behavioral disorders, such as self-mutilation, also became evident. She was diagnosed with complex sleep apnea syndrome and noninvasive bi-level positive airway pressure was introduced. Repeated respiratory infection, which frequently caused hypoventilation while she was asleep, triggered the need for permanent nocturnal PPV. Self-dislodgement of the ventilator tube associated with her mental retardation led to hypoxic encephalopathy. Tracheotomy was performed at age 4 years for chronic home mechanical ventilation. Though progressive hypoventilation extended into the daytime, the patient refused ventilator support and managed disconnection of the respiratory circuit when she was awake. Finally, the other institution gave a diagnosis of LO-CCHS. There was no response to respiratory stimulants, including theophylline and progesterone.

At the initial evaluation at our hospital, pure oxygen (0.12–0.25 L/min) was supplied through a speech cannula during the daytime if it was acceptable to her. PPV was managed during the nighttime with a Trilogy 200 Plus™ (Philips Respironics GK, Tokyo, Japan) in synchronized intermittent mandatory ventilation (SIMV) mode as follows: respiration rate of 20 breaths/min, positive end-expiratory pressure (PEEP) of 5 cm H_2_O, tidal volume of 160 mL, and inspiratory phase time of 0.8 s. Her peripheral venous blood gases on room air without ventilator support were pH 7.1, PO_2_ 24 mmHg, PCO_2_ 116 mmHg, and HCO_3_ 41.9 mmol/L. She showed neither carbon dioxide narcosis nor overt respiratory distress even though her EtCO_2_ level was 60–85 mmHg. Preoperative computed tomography revealed a posterior mediastinal tumor, which was suspected to have originated from the sympathetic nervous system, such as neuroblastoma or ganglioneuroma. Because this tumor did not compress the pulmonary artery or trachea, and thus was not considered to have affected her hemodynamics or respiratory status, further investigation has not been performed before operation. Echocardiography confirmed that her cardiac function was preserved. Additionally, lactic acidosis and 3-hydroxy-3-methylglutaric acidemia, which suggest potential mitochondrial dysfunction, were observed at a preoperative examination, but definitive diagnoses were not obtained. After approval by the ethics committee at our institution, a multidisciplinary conference was held to discuss the perioperative strategy for our patient. Based on a consideration of her mental status, mechanical ventilation under minimum sedation during the early postoperative period was deemed to be required to stabilize the surgical site. She was classified as American Society of Anesthesiologists Physical Status Class 3.

### Anesthesia

No premedication was given. On the day of the operation, the patient received her usual morning doses of valproic acid (200 mg) and levocarnitine (100 mg). She arrived at the operating room in a wheelchair without a ventilator. The standard American Society of Anesthesiologists monitoring devices were attached, and inhalation was induced via the tracheostomy using an increasing concentration of sevoflurane. Atropine (0.3 mg), midazolam (1 mg), fentanyl (30 μg), and rocuronium (20 mg) were given. The tracheotomy tube was then replaced by an endotracheal tube to facilitate surgical intervention. General anesthesia was maintained with sevoflurane and remifentanil. Acetate Ringer solution containing 5% dextrose was prepared to prevent hypoglycemia and lactic acidosis; the blood glucose level was 181–192 mg/dL, and the lactate level was 1.3–3.5 mmol/L. Volume control ventilation (VCV) with a tidal volume of 120 mL and a respiratory rate of 14 breaths/min was adopted to achieve an EtCO_2_ level over 45 mmHg.

The operative procedure was based on a previous report of diaphragm pacing by stimulation of the phrenic nerve with a spinal cord stimulator in patients with central hypoventilation syndrome [8]. Linear horizontal skin incisions about 3 cm long were made bilaterally 2 cm rostral to the clavicle, crossing the posterior border of the sternocleidomastoid muscle (SCM). After the dissection of subcutaneous tissue and medial retraction of the SCM, sugammadex (80 mg) was given for phrenic nerve identification. The anterior scalene muscle and the phrenic nerve were identified over the scalene muscle by monopolar electrical stimulation (5 Hz, 0.1-ms pulse width, 1–10 V) with muscle contraction of the diaphragm. The nerve sheath of the phrenic nerve was carefully dissected about 1 cm in length. A quadri-polar stimulating electrode was placed across the phrenic nerve and fixed with sutures to the surrounding connective tissue. Two seconds of phrenic nerve stimulation could induce a tidal volume of about 120 mL. An implanted pulse generator (IPG) (Brio Dual 8; Abbott Laboratories, Chicago, IL, USA) was placed under the fascia of the right abdominal rectus muscle. The generator and electrodes were connected by extension leads via subcutaneous tunnels. Approximately 2 h before the end of surgery, dexmedetomidine infusion was commenced at 6 μg/kg/h, which was changed to 0.7 μg/kg/h after 10 min. Intravenous acetaminophen (600 mg) and infiltration analgesia with a total of 6.8 mL of 0.75% ropivacaine were also given. At the end of anesthesia, the patient did not show any clinical signs of emergence. Dexmedetomidine infusion was decreased to 0.4 μg/kg/h. Blood pressure and heart rate remained within 20% of the baseline level without pharmacological manipulation.

### Post-operative course

**Fig. 1:**
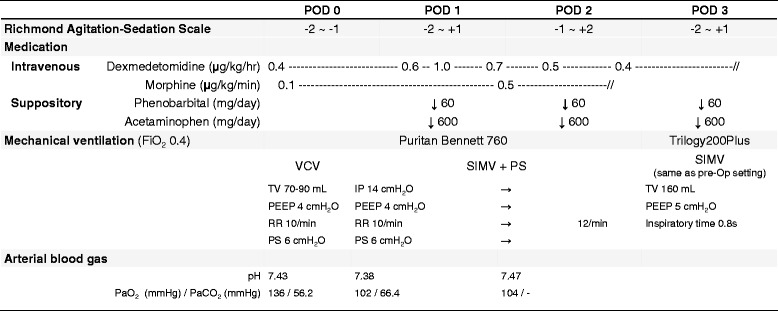
Post-operative course. Post-operative course of her medication and respiratory support

The patient showed body movement in response to verbal stimulation when she arrived in the intensive care unit (ICU). Continuous intravenous infusion of morphine was commenced at 0.1 μg/kg/min. Then, postoperative sedation and pain control were managed by combining dexmedetomidine (0.4–1.0 μg/kg/h) and morphine (0.1–0.5 μg/kg/min) to achieve a score on the Richmond Agitation-Sedation Scale of from −1 to −2. She was initially placed on mechanical ventilation with a Puritan Bennett™ 760 (Medtronic, Minneapolis, MN, USA) in VCV, but this was subsequently changed to SIMV with pressure support mode as follows: inspiratory pressure of 14 cm H_2_O, PEEP of 4 cm H_2_O, respiration rate of 10 breaths/min, and pressure support of 6 cm H_2_O. An arterial blood gas analysis performed with FiO_2_ at 0.4 on the ventilator showed pH 7.38, PaO_2_ 102 mmHg, PaCO_2_ 66 mmHg, and HCO_3_ 39 mmol/L. Intravenous medications were gradually replaced by phenobarbital and acetaminophen suppositories from postoperative day (POD) 1. Since postoperative pain and hemostasis were confirmed to be well controlled, phrenic nerve stimulation was tried on POD 3. To avoid fatigue of the phrenic nerve and diaphragm, we applied unilateral stimulation (2 s on/4 s off) for 30 min on each side: 3.5 mA to the left phrenic nerve and 3.0 mA to the right phrenic nerve. The level of EtCO_2_ subsequently decreased from over 80 to 60 mmHg. Because the patient was able to tolerate phrenic nerve stimulation without overt respiratory distress, intravenous morphine was discontinued and a Trilogy 200 Plus™ was reintroduced with her preoperative settings. Following monitoring of the blood concentration of phenobarbital, dexmedetomidine infusion was tapered. On POD 3, dexmedetomidine was terminated and the patient was discharged from ICU with a home ventilator for pacemaker adjustment and management [Fig. 1]. Since it gradually became impossible to achieve sufficient ventilation by left phrenic nerve stimulation, probably due to stimulator detachment, the output strength of the IPG for right phrenic nerve stimulation and the duration of stimulation were managed to achieve a target EtCO_2_ level of 50–60 mmHg. On POD 17, the daily dose of suppository phenobarbital was increased from 60 to 75 mg to attenuate nociception for pacemaker adaptation. Since phrenic nerve stimulation provided substantially physiological sleep, the patient showed an increase in appetite. Her mother also showed reduced levels of physical and psychological stress. On POD 19, the patient was discharged from the hospital with a final setting for stimulation of the right phrenic nerve of 2.5 mA. Because the cost of the device is not reimbursed by the health care insurance system in Japan, all medical expenses were covered by the patient’s family.

### Discussion

This case highlights that the perioperative management of CCHS must be modified based on coexisting diseases, since CCHS includes several specific issues in various organ systems. Due to the autonomic dysregulation caused by CCHS itself and potential mitochondrial dysfunction, we used sevoflurane instead of propofol and gave a lactate-free infusion containing 5% dextrose under blood glucose monitoring to avoid perioperative hypoglycemia and lactic acidosis [9, 10].

There are no guidelines or randomized trials to identify the optimal anesthetic approach in CCHS. According to the latest systematic review by Basu et al., there have been few reports on the use of anesthesia in patients with established CCHS [1]. In fact, little is known even about the use of muscle relaxants and their impact on postoperative apnea. It is recommended that, if neuromuscular blockade is considered to facilitate surgical intervention, neuromuscular blockade should be monitored and sugammadex is needed to avoid residual blockade [1]. In the present case, the tracheostomy tube was replaced by orotracheal intubation because surgical intervention that reaches the cervical region may cause accidental extubation of the former. Moreover, early extubation was not required as postoperative respiratory support was planned in advance. In addition, relatively short-acting anesthetic agents were preferred to minimize postoperative respiratory depression. Drugs that directly decrease blood pressure and heart rate are not recommended since CCHS is a multisystem disorder that causes autonomic instability, resulting in possible bradycardia and transient asystole [9].

Dexmedetomidine, an alpha-2 agonist which has a sedative effect by reducing the activity in the locus coeruleus in the central nervous system, has been used as a perioperative adjunct drug to reduce complications such as agitation, delirium, shivering, and pain in pediatric patients [11]. Although minimal depression of the respiratory system while maintaining a patent airway is a considerable advantage of dexmedetomidine, there have been few reports of its use in CCHS. Kameyama et al. described a 2-year-old girl with CCHS who required postoperative mechanical ventilation due to poor intraoperative oxygenation. The co-administration of dexmedetomidine and midazolam was selected to avoid continuous sedation. However, they did not suspend sedative infusions because the patient became agitated [12]. This suggests that a combination of dexmedetomidine and midazolam is insufficient to attenuate nociception caused by artificial ventilation. Thus, in our case, low-dose intravenous morphine was supplemented with dexmedetomidine to attenuate the levels of pain and nociception. Morphine is not a short-acting agent and potentially causes respiratory depression if an overdose is given, but we estimated that several days would be required for pacemaker adjustment and adaptation. Therefore, we chose low-dose intravenous morphine as a reliable analgesia. It has been reported that opioid-free continuous paravertebral block provided appropriate analgesia with less respiratory depression [13]. However, the surgical site of our patient extended to the abdomen, and her history of repeated self-dislodgement of the ventilator tube suggested the possibility of accidental catheter removal. The mechanism of the analgesic effect of dexmedetomidine is still unclear and may be partly due to its function as an anxiolytic. The ability to reduce opioid consumption through the use of dexmedetomidine is an advantage for patients with CCHS. Our patient seemed to be well-sedated with a combination of dexmedetomidine (1.0 μg/kg/h) and morphine (0.1 μg/kg/min), but she was easily aroused and responded to nursing intervention. When dexmedetomidine was decreased to 0.7 μg/kg/h, spontaneous ventilation was regained. Dexmedetomidine has not been extensively studied in terms of its pharmacologic and physiologic effects in a pediatric population [11]. Therefore, we initiated dexmedetomidine infusion beginning in the intraoperative period under strict observation and monitoring by anesthesiologists. We observed neither cardiac instability caused by loading infusion nor inadequate sedation during patient transfer.

## Conclusions

Patients with CCHS can have systemic comorbidities, and the obvious concern to anesthesiologists is the defective autonomic control of ventilation, which may cause postoperative complications. In the present case, a combination of intravenous dexmedetomidine and morphine provided effective conscious sedation with protection of the surgical site even when CCHS was the known diagnosis.

## Abbreviations

CCHS: Congenital central hypoventilation syndrome
ICU: Intensive care unit
IPG: Implanted pulse generator
LO-CCHS: Late-onset congenital central hypoventilation syndrome
PEEP: Positive end-expiratory pressure
POD: Postoperative day
PPV: Positive pressure ventilation
SCM: Sternocleidomastoid muscle
SIMV: Synchronized intermittent mandatory ventilation
VCV: Volume control ventilation
